# Retraction: A Rare Surgical Case of Isolated Double-Chambered Right Ventricle Without Cardiac Malformations in an Older Female Patient

**DOI:** 10.7759/cureus.r162

**Published:** 2025-01-22

**Authors:** Yuki Hayashi, Shunji Osaka, Satoshi Unosawa, Masashi Tanaka

**Affiliations:** 1 Cardiovascular Surgery, Nihon University School of Medicine, Tokyo, JPN; 2 Cardiovascular Surgery, Akabane Central General Hospital, Tokyo, JPN; 3 Cardiovascular Surgery, Unosawa Clinic, Chiba, JPN

This article has been retracted by the Editors-in-Chief due to duplicate publication. The submitting author's supervisor had previously submitted and published this case in another journal: https://journals.sagepub.com/doi/10.1177/0218492317714667. The submitting author contacted Cureus to request retraction upon discovery of this prior publication.

